# The assessment of atlantoaxial joint involvement in patients with rheumatoid arthritis, results from an observational “real-life” study

**DOI:** 10.1038/s41598-023-46069-0

**Published:** 2023-11-17

**Authors:** Claudia Di Muzio, Alessandro Conforti, Federico Bruno, Damiano Currado, Onorina Berardicurti, Luca Navarini, Viktoriya Pavlych, Ilenia Di Cola, Alice Biaggi, Stefano Di Donato, Annalisa Marino, Sebastiano Lorusso, Francesco Ursini, Antonio Barile, Carlo Masciocchi, Paola Cipriani, Roberto Giacomelli, Piero Ruscitti

**Affiliations:** 1 Rheumatology Unit, Department of Biotechnological and Applied Clinical Sciences, University of L’Aquila, Delta 6 Building, PO Box 67100, L’Aquila, Italy; 2 Clinical and research section of Rheumatology and Clinical Immunology, Fondazione Policlinico Campus Bio-Medico, Via Álvaro del Portillo 200, 00128 Rome, Italy; 3 Rheumatology Unit, IRCCS Istituto Ortopedico Rizzoli, SSD Medicina e Reumatologia, 40136 Bologna, Italy; 4 Department of Biomedical and Neuromotor Sciences (DIBINEM), Alma Mater Studiorum, University of Bologna, Bologna, Italy; 5 Present Address: Rheumatology and Clinical Immunology, Department of Medicine, University of Rome “Campus Biomedico”, School of Medicine, Rome, Italy

**Keywords:** Musculoskeletal system, Rheumatology, Rheumatic diseases, Rheumatoid arthritis

## Abstract

Atlantoaxial joint is a possible affected site during rheumatoid arthritis (RA) and, in this work, we evaluated its occurrence and associated characteristics in a “real-life” cohort. By a medical records review study of RA patients longitudinally followed-up, the occurrence of severe atlantoaxial joint involvement was estimated (incidence proportion and incidence rate per 1000 person-years at risk). Regression analyses were also exploited to evaluate possible associated factors. Based on these findings, a prospective recruitment was performed to build a descriptive cross-sectional study in evaluating a subclinical atlantoaxial joint involvement in patients with the same clinical characteristics. Retrospectively, 717 patients (female 56.6%, age 64.7 ± 12.3 years) were studied. The incidence proportion of severe atlantoaxial joint involvement was 2.1% [1.5–2.5], occurring in 15 out of 717 patients, and identified by both MRI and CT scan. Considering over 3091 person-years, an incidence rate of 5.2 × 1000 [2.9–8.3] person-years was estimated. Regression analyses suggested that male gender, a longer disease duration, ACPA positivity and extra-articular manifestations resulted to be significantly associated with a severe atlantoaxial joint involvement. Given these findings, 30 asymptomatic patients were selected according to these clinical characteristics and underwent MRI of cervical spine. To date, almost 50% of these asymptomatic patients showed a subclinical atlantoaxial joint involvement. The occurrence of the severe atlantoaxial joint involvement in RA patients was estimated in a “real-life” setting. Male gender, ACPA positivity, long disease duration, and extra-articular manifestations could be associated with the severe atlantoaxial joint involvement in RA. MRI could provide a useful clinical tool to early evaluate the atlantoaxial joint involvement in RA, also in asymptomatic patients.

## Introduction

Rheumatoid arthritis (RA) is a systemic inflammatory disease associated with a significant morbidity and mortality^1,2^. Clinically, RA manifests as a chronic, symmetrical articular disease, typically affecting the small joints, typically proximal interphalangeal and metacarpophalangeal joints^1^. However, any synovial joint may be involved, and the cervical spine may be another possible affected site of the disease^1^. As early as 1890, Garrod AE reported the first description of 178 patients with cervical spine involvement in a series of 500 patients with RA, reporting a prevalence around 30%^3^. Subsequently, many works investigated this issue with different and partially conflicting results^4–11^, highlighting the need of further studies to fully elucidate this topic. In fact, a highly different prevalence of cervical spine involvement is reported in RA patients, ranging between 25 and 88%^4–8^. During RA, the atlas-axis cervical vertebrae 1 and 2 (C1 and C2) articulation may be typically involved, between the transverse ligaments of the atlas and the posterior side of the odontoid. At this level, hypertrophic synovitis, juxta-articular bone erosions, joint ankylosis, and local osteoporosis may induce a late spinal instability, especially when involving the transverse ligament provoking a ligamentous laxity and a consequent cranial migration of the odontoid^4–6^. From a clinical point of view, in initial stages, patients may be asymptomatic; usually, the neck pain is the presentation of the cervical involvement in RA^5,6,9^. Subsequently, the compression of the cranial nerves may lead to other clinical features, such as occipital headache, migraines, and neck, mastoid, ear, or facial pain^4–7^. In later and more severe stages, patients may experience a cervical instability, generally reporting a clinical picture of crepitation associated with a sensation of their head falling forward upon flexion^5,6,8^. In this context, the diagnostic role of computed tomography (CT) and magnetic resonance imaging (MRI) has been recently highlighted in an accurate recognition of this manifestation^7,8^. A prompt identification of such feature is of importance to timely manage these patients. In fact, when the patient is still asymptomatic, a therapeutic strategy may be of crucial importance to induce a complete resolution of this feature^5,6^. Conversely, when the patient begins to complain the first symptoms of atlantoaxial joint involvement, a severe radiological picture is usually present, which often requires a more aggressive and less frequently successful treatment. However, presently, international validated recommendations are still lacking about the timing as well as the management of atlantoaxial joint involvement in RA patients. In addition, a few studies specifically focused on severe atlantoaxial joint involvement, when a clinically relevant picture and radiologic damage are present^7,9–11^. Furthermore, predictive factors are not entirely clarified to suggest patients at higher risk of developing such involvement in patients with RA.

On these bases, in this work, we aimed at evaluating the occurrence of a more severe atlantoaxial joint involvement in RA by a medical records review study in a “real-life” cohort in a first phase of the study. Furthermore, we exploited a clinical risk profile to identify some factors associated with a more severe atlantoaxial joint involvement in this first retrospective phase. After that, a prospective recruitment was performed to build a cross-sectional study to descriptively assess a possible subclinical atlantoaxial joint involvement by MRI. This second phase of the study involved asymptomatic patients but all characterised by derived associated factors from the analysis of the retrospective phase.

## Materials and methods

### Study design, setting, patients, and variables to be assessed in retrospective phase

In this study, we performed a medical records review study of consecutive patients with RA longitudinally followed-up at Rheumatologic Clinics of University of L’Aquila, L’Aquila, Italy, and of University of Campus Biomedico of Rome, Rome, Italy, between January 2010 and December 2020. Patients who met the ACR/EULAR 2010 criteria were assessed^12^. Each patient clinical chart was reviewed to assess the occurrence of a severe atlantoaxial joint involvement. The latter was defined as a clinically relevant picture in association with the presence of one or more of these radiologic features on atlantoaxial joint: large bone oedema, extensive synovitis, bone erosions, spinal cord compression, and/or atlantoaxial instability (subluxation, impaction). In addition, symptoms of atlantoaxial joint involvement were investigated and collected including occipital headache, migraines or and neck, mastoid, ear, facial pain, tinnitus, vertigo, and loss of equilibrium. In the present evaluation, these additional variables were also registered: age, gender, body mass index (BMI), smoking habit, disease duration, rheumatoid factor (RF), anticitrullinated protein antibodies (ACPA), and administered therapies. Glucocorticoids (GCs), synthetic-, biological-, and target synthetic disease modifying anti-rheumatic drugs (DMARDs) were recorded. GCs therapy was also categorised, either high dosage or low dosage, as previously reported^13^. All patients with atlantoaxial joint symptoms were investigated by both MRI and CT scan to elucidate a possible severe involvement due to RA. After that possible associated factors of severe atlantoaxial joint involvement were derived by regression analyses to exploit a clinical risk profile about this issue.

The local Ethics Committee (*Comitato Etico Azienda Sanitaria Locale 1 Avezzano/Sulmona/L'Aquila, L'Aquila*, Italy, protocol number 0095184/20) approved the study, which was performed according to the Good Clinical Practice guidelines and the Declaration of Helsinki. In reporting the results, we followed the STROBE guidelines.

### Data sources, bias, and study size in retrospective phase

Relevant data were collected by a review of clinical charts of patients attending the involved centres between 2010 and 2020. Considering the retrospective design of this analysis, we tried to minimize possible biases with a careful definition of each variable to be assessed. Patients with concomitant neurological diseases, history of cervical trauma, and previous pathologies of the cervical spine were excluded from the assessment. A specific sample size was not estimated since this would be “real-life” evaluation of atlantoaxial joint involvement in patients with RA in our two cohorts.

### Prospective recruitment of the study to build a cross-sectional study to assess a possible subclinical atlantoaxial joint involvement by MRI

**Figure 1:**
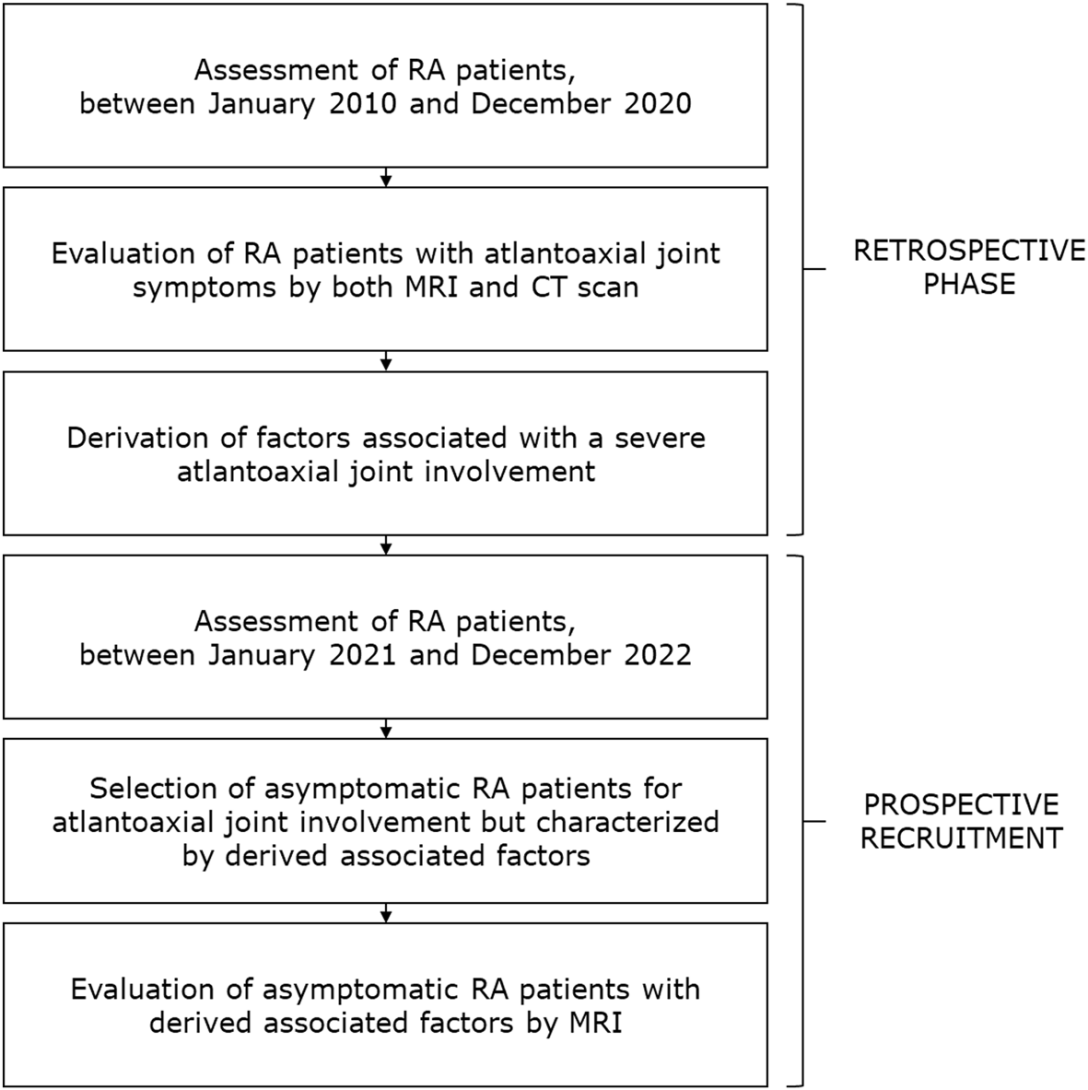
Study design; based on the retrospective section, we built a prospective phase of recruitment that served to build a cross-sectional study to assess a possible subclinical atlantoaxial joint involvement by MRI.

In addition, according to the derived associated factors with the severe atlantoaxial joint involvement in retrospective study, a prospective recruitment was carried out that served to build a cross-sectional study to assess a possible subclinical atlantoaxial joint involvement by MRI (Fig. 1). Between January 2021 and December 2022, consecutive asymptomatic RA patients, who were characterized by the same associated factors (at least 3 out of 4 derived clinical variables) obtained by our analysis, underwent a cervical MRI to evaluate a possible subclinical atlantoaxial joint involvement. This prospective recruitment was performed to have a 2:1 matching with patients with a severe atlantoaxial joint involvement, who were assessed in the retrospective phase of this study.

### MRI and CT scan definitions

In the retrospective phase, instrumental imaging evaluation of the atlantoaxial joint was performed in all patients with clinical suspicion of involvement. MRI examinations were performed on a 1.5 T scanner (Discovery MR750w, GE Healthcare), acquiring T1, T2, and STIR (short-tau inversion recovery) sequences on sagittal, coronal, and axial planes. CT examinations were performed on a multidetector 320-row CT scanner (Aquilion One, Toshiba) acquired with a thin collimation; soft tissue or bone algorithms were applied for image data reconstruction and analysis. Image analysis was performed by two expert radiologists dedicated to musculoskeletal and spine imaging. In particular, MRI images were examined to depict the presence of thickened synovium around the odontoid process, bone marrow oedema, and spinal cord changes, whereas CT images provided a superior visualization of bone erosion and atlanto-axial or sub-axial subluxation. In the prospective recruitment, the same MRI protocol was similarly used to highlight a possible subclinical atlantoaxial joint involvement. In both phases, experienced radiologists evaluated the collected images.

### Statistical methods

In retrospective phase, statistics provided descriptive analysis of the data. Normally distributed continuous variables were expressed as mean ± standard deviation (SD), otherwise as median and range interquartile (IQR), as appropriate. Patients with significant missing data were removed. In retrospective phase, occurrence of severe atlantoaxial joint involvement was also estimated by incidence proportion and incidence rate per 1000 person-years at risk. In addition, Cox regression analyses were exploited to evaluate possible associated factors of severe atlantoaxial joint involvement in this phase of our study. Based on the number of retrieved patients codified as having this feature only univariate analyses were performed following the general rule that 10 events of the outcome of interest are required for each variable in the model including the exposure of interest (i.e. 10:1 events per variable)^14^. Thus, possible “sparse data” biases, related to multivariate analysis in small cohort of patients, were minimised^15^. These univariate analyses had the main purposes to provide the basis of arranging a specific prospective recruitment in which we selected patients, who could be considered at higher risk according to these features, to assess a possible subclinical atlantoaxial joint involvement by MRI. The derived results of this second section were descriptively assessed. Similarly to the retrospective phase, normally distributed continuous variables were expressed as mean ± SD, otherwise as median and IQR.

The Statistics Package for Social Sciences (SPSS for Windows, version 17.0, SPSS Inc., Chicago, IL, USA) was used for all analyses.

### Ethics approval and consent to participate

The local Ethics Committee (Comitato Etico Azienda Sanitaria Locale 1 Avezzano/Sulmona/L’Aquila, L’Aquila, Italy; protocol number 0095184/20) approved the study, which was performed according to the Good Clinical Practice guidelines and the Declaration of Helsinki. All patients provided written informed consent to participate before data collection.

## Results

### Descriptive characteristics and incidence of severe atlantoaxial joint involvement in our cohort in the retrospective phase of the study

In this evaluation, 717 patients were assessed (Table 1), mostly female (56.6%) with a mean age of 64.7 ± 12.3 years. The median disease duration was 10 years (range interquartile 28) and 68.3% displayed the positivity for RF and/or ACPA. During the follow-up (6.2 ± 3.3 years), 78.2% of patients were treated with low dose of GCs, 51.9% with methotrexate (MTX), and 45.7% with biologic DMARDs. Moreover, 48 out of 717 (6.7%) had extra-articular manifestations, 26 patients were affected by a secondary Sjogren’s syndrome. Interstitial lung disease (ILD) was identified in 16 patients whereas 3 had a leukocytoclastic vasculitis. Finally, 3 patients had rheumatoid nodules.

**Table 1:** Demographic and clinical features of the evaluated patients in retrospective phase.

	Whole cohort, retrospective phase	Patients with atlantoaxial joint involvement, retrospective phase
Patients	717	15
Gender (female/male)	56.6%/43.4%	87.7%/13.3%
Age, mean ± SD, years	64.7 ± 12.3	58.0 ± 11.8
Disease duration at the first observation, median (range), years	10 (28)	6 (7)
< 1 year	40.6%	40.0%
Between 1 and 5 years	34.0%	33.3%
Between 5 and 10 years	25.4%	26.7%
RF and/or ACPA	68.3%	100%
Extra-articular disease	6.7%	40.0%
Smoking habit	34.4%	46.6%
GCs low dose	78.2%	0.0%
GCs high dose	12.6%	100.0%
MTX	71.9%	100.0%
HCQ	25.9%	14.2%
LEF	13.1%	0.0%
SSZ	7.1%	0.0%
Biologic DMARDs	43.5%	60.0%
TNFis	26.2%	60.0%
Non-TNFis	17.3%	0.0%
Targeted synthetic DMARDs	2.5%	0.0%

*RF* rheumatoid factor, *ACPA* anticitrullinated protein antibodies, *GCs* glucocorticoids, *MTX* methotrexate, *HCQ* hydroxychloroquine, *LEF* leflunomide, *SSZ* sulfasalazine, *DMARD* disease-modifying anti-rheumatic drug, *TNFis* tumor necrosis factor.

In this cohort, the cumulative incidence proportion of severe atlantoaxial joint involvement was 2.1% [1.5–2.5], occurring in 15 out of 717 patients. Considering over 3019 person-years, an incidence rate of 5.2 × 1000 [2.9 − 8.3] person-years was also estimated.

### Characteristics of patients with atlantoaxial joint involvement in the retrospective phase of the study

The mean age of 15 patients with atlantoaxial joint involvement was 64.7 ± 12.3 years and most patients were female, 7 reported a smoking habit. At the beginning, all these patients experienced a mild pain of the cervical spine with an insidious onset. After that, over few weeks, out of 15 patients, 10 become strongly symptomatic, experiencing an intense neck pain, 3 reported vertigos, 2 described loss of equilibrium, and 1 complained tinnitus. The atlantoaxial joint involvement was confirmed by both MRI and CT scan.

**Figure 2:**
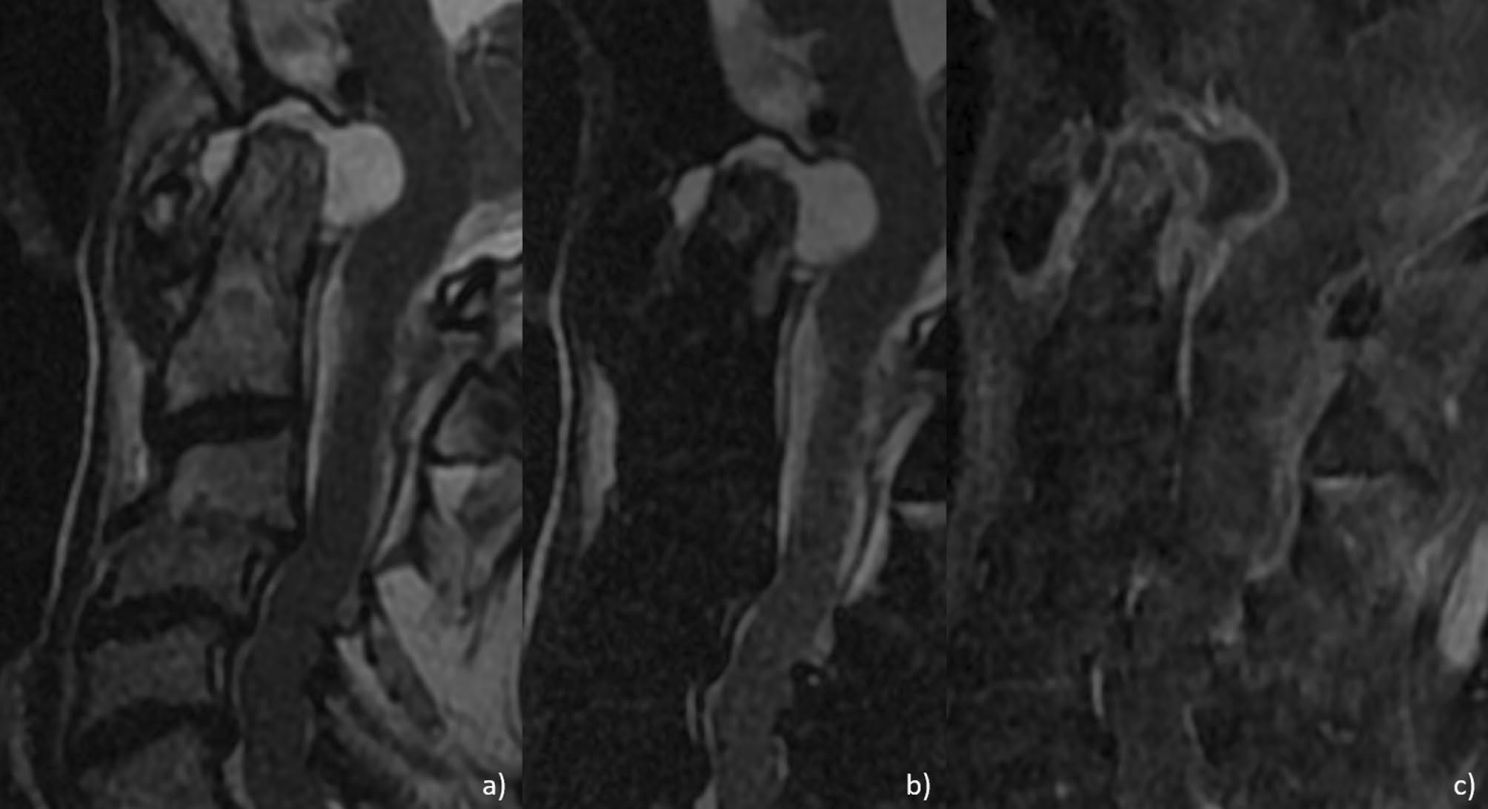
Sagittal T2 (**a**), fat-saturated T2 (**b**) and contrast enhanced T1 (**c**) MRI images showing marked synovial hypertrophy and effusion, with edema and erosions of the odontoid peg.

**Figure 3:**
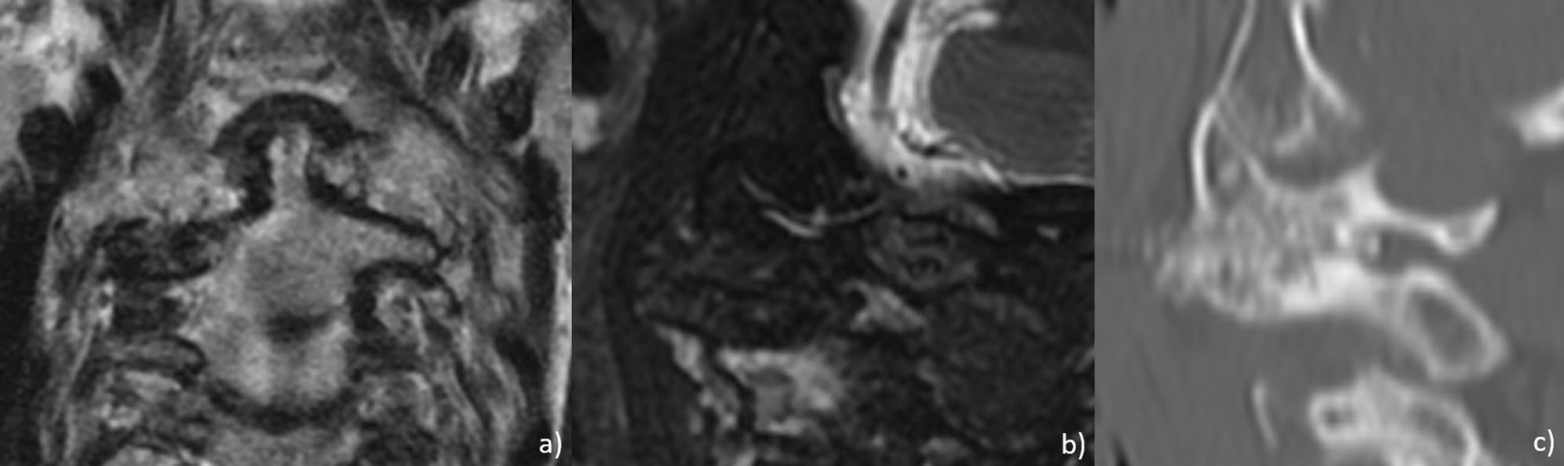
Coronal T2 (**a**), sagittal fat-saturated T2 (**b**) and sagittal CT (**c**) MRI images showing erosion and partial fusion of left lateral atlantoaxial joint.

**Figure 4:**
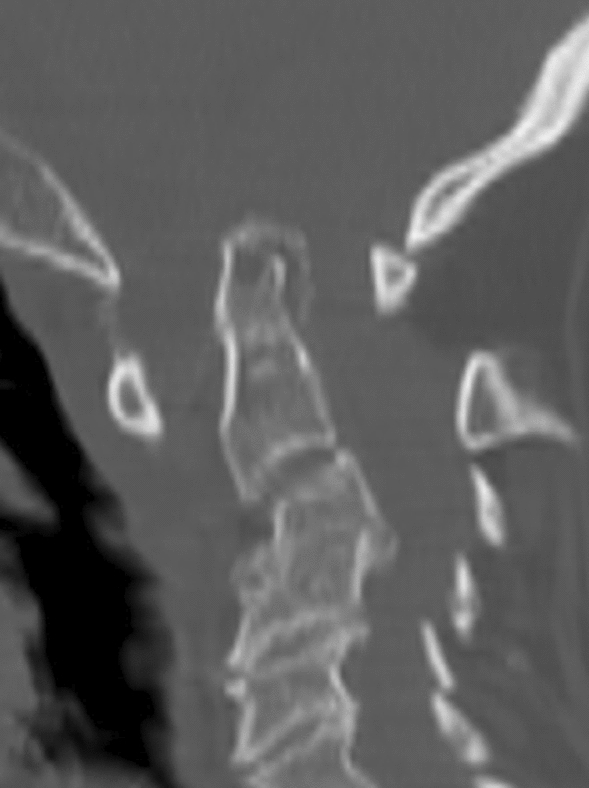
Sagittal CT reconstruction depicting several findings consistent with increased anterior atlantoaxial distance and cephalad migration of C2 (cranial settling), findings consistent with atlantoaxial subluxation and atlantoaxial impaction.

MRI images reported a synovial hypertrophy in 100% of these patients, whereas 86.7% showed a spinal cord compression (Fig. 2). The presence of bone oedema was recognized in 73.3% of patients, and 53.3% were characterized by bone erosions, confirmed by CT scan (Fig. 3). Out of assessed patients, 2 also showed cervical instability (Fig. 4). The presence of ACPA was reported in all these patients. None of these patients was in clinical remission at the time of diagnosis of severe atlantoaxial joint involvement onset. Furthermore, this manifestation occurred within a mean of 5 years from the onset of the disease, and 10 out of 15 had were previously treated with short and low doses of GCs whereas 11 patients were treated with synthetic DMARDs and 3 with biologic DMARDs. Extra-articular manifestations were associated with severe atlantoaxial joint involvement, 4 patients presented ILD and 2 presented rheumatoid nodules. None of these patients reported a previous cervical trauma. After the diagnosis of severe atlantoaxial joint involvement, all these patients were treated with MTX and high doses of intravenous GCs, 9 out of 15 were also treated with TNF inhibitors (TNFi) (7 infliximab, 1 certolizumab-pegol, 1 etanercept). This therapeutic strategy resulted in a complete resolution of the inflammatory synovitis in all but two patients. These developed a cervical spinal instability resulting in neurological progression and required a subsequent surgical management.

### Clinical factors associated with of severe atlantoaxial joint involvement in our cohort of patients in retrospective phase of the study

As shown in Table 2, considering the relatively low incidence of severe atlantoaxial joint involvement in our study, only univariate explorative HR analyses were exploited to assess possible factors associated with such manifestation in this retrospective phase of the study. Male gender (HR 10.97, 95% CI 2.47–48.81, p = 0.002) and a longer disease duration (HR 1.06, 95% CI 1.01–1.10, p = 0.022) resulted to be significantly associated with a severe atlantoaxial joint involvement. Similarly, ACPA positivity (HR 4.87, 95% CI 1.10–21.60, p = 0.037) and extra-articular manifestations (HR 7.83, 95% CI 2.81–21.81, p < 0.0001) were associated with a severe atlantoaxial joint involvement in our cohort in this retrospective phase of the study.

**Table 2:** Cox regression univariate analysis: predictors of severe atlantoaxial joint involvement in our cohort in retrospective phase of the study.

Clinical variables	HR	95% CI	p-values
Age	0.98	0.95–1.02	0.020
Male gender	10.97	2.48–48.81	**0.002**
Smoking habit	1.36	0.83–10.93	0.566
ACPA	4.87	1.10–21.60	**0.037**
RF	3.92	0.85–17.40	0.072
Disease duration	1.06	1.01–1.10	**0.022**
Extra-articular disease	7.83	2.81–21.81	**< 0.001**
Surgery	2.80	0.66–6.61	0.580
MTX	0.52	0.30–2.27	0.500
TNFi	2.28	0.81–6.38	0.900
Non-TNFi	0.95	0.25–3.21	0.903

*ACPA* anticitrullinated protein antibodies, *RF* rheumatoid factor, *MTX* methotrexate, *TNFis* tumor necrosis factor.

Significant values are in bold.

### Clinical features and results obtained in the prospective recruitment of the study

In the prospective recruitment of this work, 30 consecutively asymptomatic RA patients were selected and assessed among those attending the involved centres to build a cross-sectional study in evaluating a possible subclinical atlantoaxial joint involvement by MRI (Table 3). They were characterized by at least 3 of the 4 associated factors identified by regression analyses in the retrospective phase. Specifically, they were mostly females (80.0%), with a mean age of 62.8 ± 11.7 years, and 33.3% of them had a smoking habit. The positivity for RF and/or ACPA was recognised in 90.0% of these patients, long disease duration in 80.0%, and extra-articular manifestations in 66.7%. At the time of assessment, 56.7% of patients were treated with low dose of GCs, 46.7% with MTX, and 76.7% with biologic DMARDs.

**Table 3:** Demographic and clinical features of the patients involved in prospective recruitment.

	Whole cohort, prospective recruitment	Patients with atlantoaxial joint involvement, prospective recruitment
Patients	30	13
Gender (female/male)	80.0%/20.0%	76.9%/23.1%
Age, mean ± SD, years	62.8 ± 11.7	64.7 ± 11.5
Disease duration at the first observation, median (range), years	10 (36)	10 (24)
< 1 year	10.0%	7.7%
Between 1 and 5 years	10.0%	7.7%
Between 5 and 10 years	80.0%	84.6%
RF and/or ACPA	90.0%	84.6%
Extra-articular disease	66.7%	69.2%
Smoking habit	33.3%	30.8%
GCs low dose	56.7%	76.9%
GCs high dose	0.0%	0.0%
MTX	46.7%	53.9%
HCQ	10.0%	15.4%
LEF	10.0%	15.4%
SSZ	6.7%	0.0%
Biologic DMARDs	76.7%	76.9%
TNFis	10.0%	7.7%
Non-TNFis	66.7%	69.2%
Targeted synthetic DMARDs	13.3%	7.7%

*RF* rheumatoid factor, *ACPA* anticitrullinated protein antibodies, *GCs* glucocorticoids, *MTX* methotrexate, *HCQ* hydroxychloroquine, *LEF* leflunomide, *SSZ* sulfasalazine, *DMARD* disease-modifying anti-rheumatic drug, *TNFis* tumor necrosis factor.

**Figure 5:**
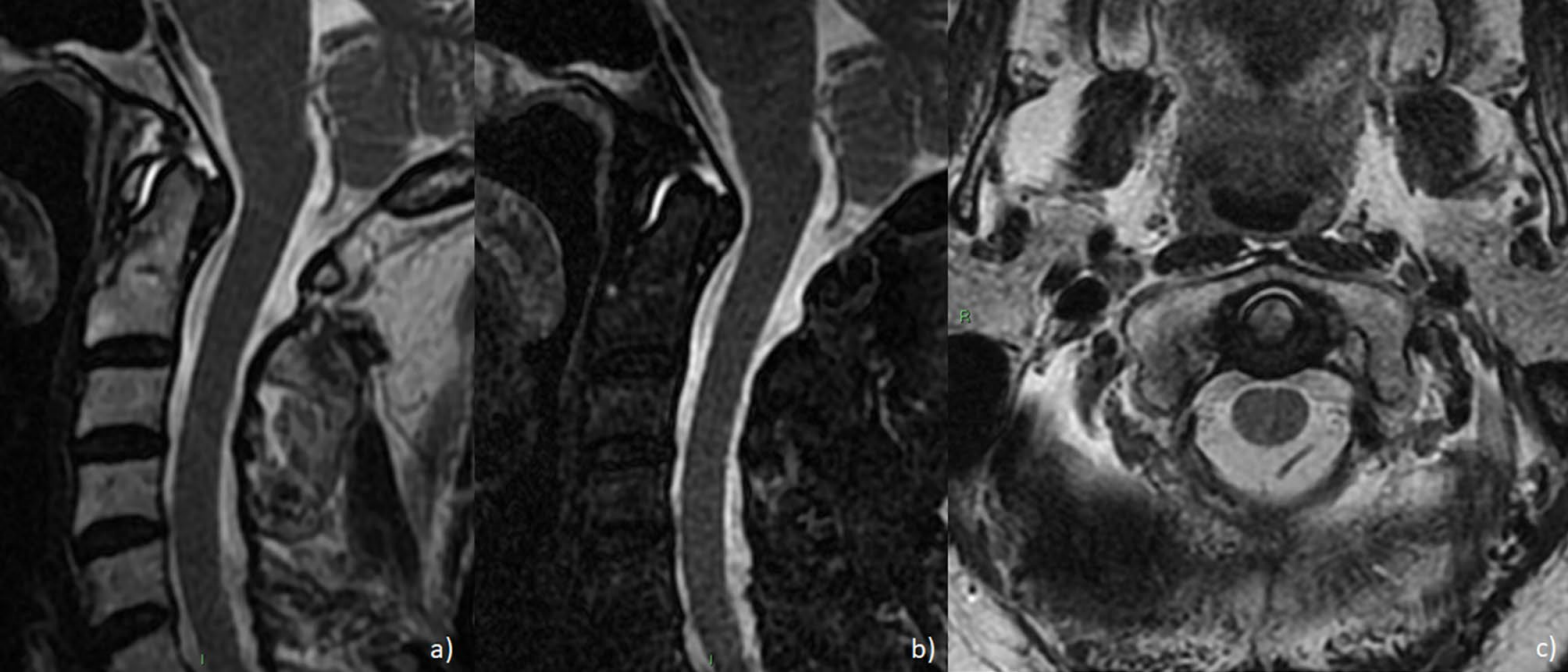
Sagittal T2 (**a**), sagittal fat-saturated T2 (**b**) and axial T2 (**c**) depicting periodontoid joint fluid with posterior thickening of the transverse ligament.

**Figure 6:**
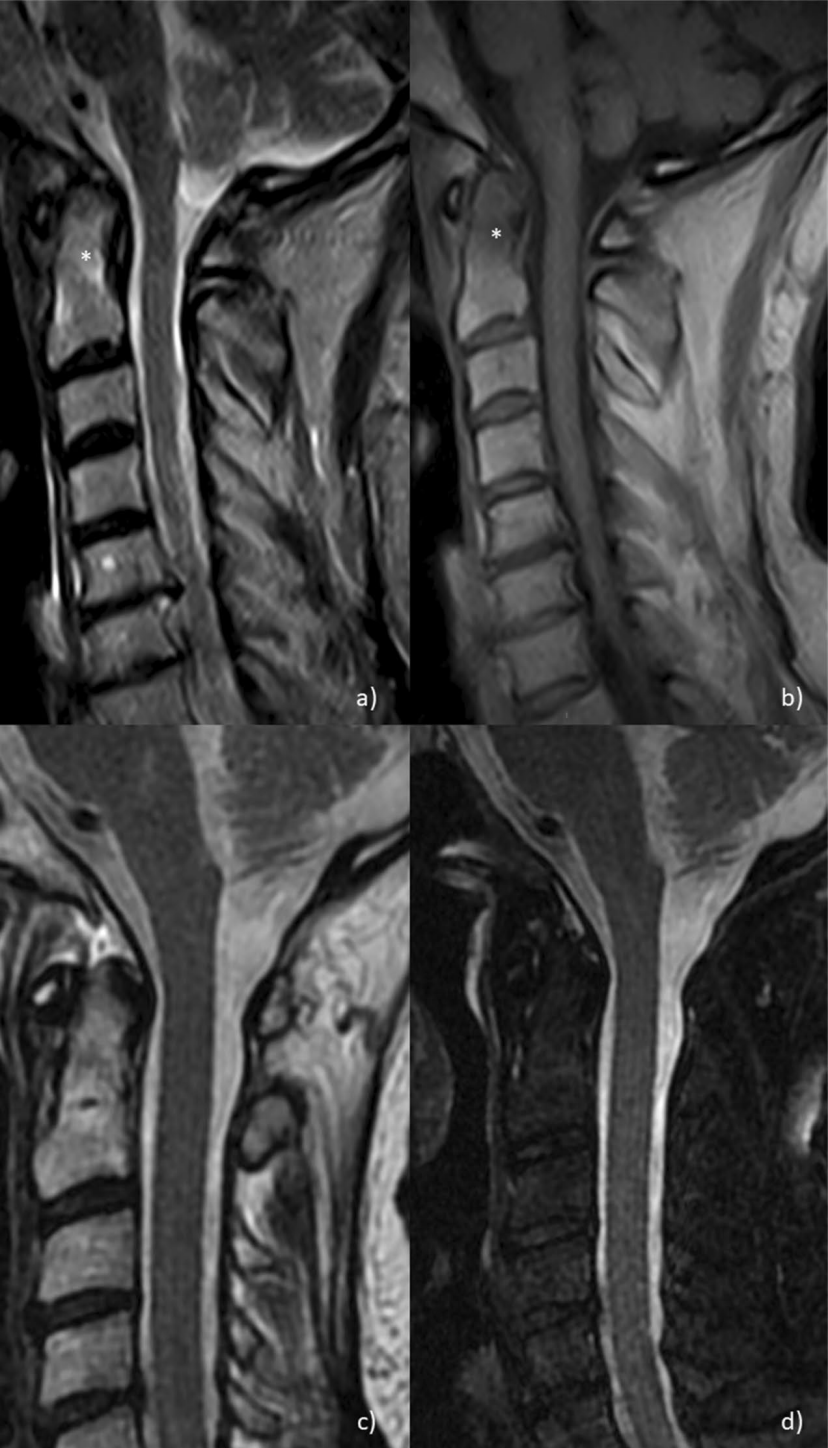
Sagittal T2 (**a**) and sagittal T1 (**b**) MRI images showing slight bone marrow edema of the odontoid peg (asterisk). Sagittal T2 (**c**), sagittal fat-saturated T2 (**d**) MRI images showing normal imaging findings at the level of the craniocervical junction.

Out of 30, 13 patients (43.3%) showed a subclinical atlantoaxial joint involvement documented by MRI. A synovial hypertrophy was reported in 100% of these patients, even if a milder involvement than previously reported in the retrospective phase (Fig. 5). Furthermore, MRI showed that 26.7% had bone oedema (Fig. 6), 13.3% a spinal cord compression, and 6.7% bone erosions. When compared with retrospective phase, a lower percentage of these MRI findings was reported in these patients. Among these patients with subclinical atlantoaxial joint involvement, 69.2% were characterized by extra-articular manifestations and 84.6% showed positivity for RF and/or ACPA. These RA patients with subclinical atlantoaxial joint involvement, although asymptomatic, were immediately evaluated for a possible change of therapeutic strategies. Based on MRI findings, the dose of daily GCs was increased, and, in biologic DMARD treated patients, a possible switch to another biologic agent was considered.

## Discussion

In our study, in the retrospective phase, the occurrence of a severe atlantoaxial joint involvement in RA patients was estimated in a “real-life” setting with an incidence proportion of 2.1% (1.5–2.5%) and an incidence rate of 5.2 × 1000 (2.9–8.3) person-years, respectively. Male gender, a longer disease duration, presence of ACPA, and extra-articular manifestations resulted to be associated with such severe manifestation in this first phase the study. Subsequently, in 30 asymptomatic RA patients, who were characterized by these clinical features, a subclinical atlantoaxial joint involvement was found in almost half of these patients in the second cross-sectional descriptive phase.

In the retrospective phase of this study, a severe atlantoaxial joint involvement was identified following the onset of mild cervical pain, with a subtle symptomatology at the beginning, which subsequently evolved, over few weeks, in a more severe clinical picture. In this setting, MRI and CT scan showed to be sensitive and specific imaging modalities for an accurate determination of an atlantoaxial joint involvement in RA^7,16,17^. Comparing our results with previous experiences^3,5,7,9,18,19^, we retrieved a lower occurrence of severe atlantoaxial joint involvement in patients with RA. This possible discrepancy with our results could be attributed to different study designs and target populations. In fact, we focused our study on more severe patients with a high suspicion clinical picture of atlantoaxial joint involvement. In our cohort, the factors associated with a severe atlantoaxial joint involvement were also exploratively assessed. Our analysis suggested that male gender could predict the occurrence of this feature, as reported in previous studies^5,7,9,18^. Although conflicting available results, a less favourable RA outcome in men could be observed^20–22^. Furthermore, the presence of ACPA was also associated with atlantoaxial joint involvement. Generally, ACPA positivity is associated with the extent of joint destruction and with a more destructive RA course^23^. Furthermore, synovial fluid from RA joints may contain citrullinated proteins, which in turn would increase the local inflammation, correlating with radiographic damage and progression^24^. In our patients, a longer disease duration was also associated with the severe atlantoaxial joint occurrence, which could be related with a longer exposition to an active inflammatory process. Long-standing RA could less likely respond to the treatment, even the administration of biological DMARDs could not completely suppress the disease activity in a large percentage of patients^24^. Thus, a long duration of the disease could influence the presence of atlantoaxial joint involvement due to a persistent inflammation over time^25^. In addition, atlantoaxial joint involvement was also associated with the presence of extra-articular manifestations in our patients. These features are related to poor long-term outcomes^25–27^. Taking together all these observations, patients with atlantoaxial joint involvement could be considered as having a more severe subset of RA^2,7,9^.

In the descriptive cross-sectional phase of this study , we evaluated the subclinical atlantoaxial involvement in asymptomatic consecutive RA patients, characterized by the associated factors identified in the retrospective part. Among asymptomatic patients who underwent MRI, almost half showed a subclinical atlantoaxial joint involvement, pointing out some clinically silent imaging abnormalities which could anticipate the occurrence of a severe clinical picture. Thus, a very early recognition of the atlantoaxial joint involvement may be allowed by MRI. The latter is a non-ionizing imaging technique with an excellent soft-tissue visualisation, a multiplanar large anatomical coverage, and it may allow a possible centralised reading^28^. Furthermore, MRI may accurately visualise and evaluate in detail soft tissue changes of synovitis, which are not detectable by conventional clinical assessment and standard radiographic methods^29–31^. Importantly, MRI is a safe technique, without ionising radiations and no risk increase of malignancies. Taking together these observations, a sophisticated imaging modality of atlantoaxial joint involvement may detect clinically silent lesions, identifying those features of the preclinical phases which could be completely reversed by an appropriate therapeutic strategy. Based on these findings, further studies may be held to evaluate the evolution of the atlantoaxial joint involvement and the most appropriate management of these patients. Exploratively comparing MRI results of the retrospective and the prospective phases of our study, we observed that these patients were similarly characterized by a synovial hypertrophy in the atlantoaxial joint. However, in patients with subclinical atlantoaxial joint involvement, a less prominent synovial involvement and with a less percentage of spinal cord compression were observed. In addition, in these patients with subclinical atlantoaxial joint involvement a lower percentage of bone oedema and bone erosions was observed. Thus, the occurrence of these MRI features may be associated with the development of the clinical picture, associated with severe atlantoaxial joint involvement. These findings parallel what observed in peripheral joints, when the appearance of bone erosions is a marker of a more progressive disease and joint damage^32^.

As far as the treatment of atlantoaxial joint involvement is concerned, conflicting information is available without specific recommendations. In any case, the early recognition of the atlantoaxial joint involvement in RA may allow the clinicians to administer an appropriate and aggressive therapeutic strategy, considering that it may cause a severe neurologic damage^9,13,16^. A combination therapy between GCs and csDMARDs, or csDMARDs and biologic DMARDs, mainly infliximab, is suggested in reducing the development as well as the progression of cervical spine lesions in RA patients^33–35^. In this context, also the administration of high dosage of GCs is also proposed to manage more severe patients^34^, even if a possible confounding by indication bias may be reported in some studies^4,18,36^. Taking together all these observations, patients with atlantoaxial joint involvement may need a timely management devoted to an early recognition and a properly inflammation reversal as the major therapeutic target to be achieved. In addition, RA patients with a severe atlantoaxial joint involvement should be considered for a possible surgical management of the associated cervical instability^5,6,8,9^. Since these patients are often treated with a combination of immunosuppressive agents, they should be carefully evaluated to reduce the risk of serious post-operative infections^37^. In any case, it must be pointed out that further studies are required to elucidate a safe therapeutic immunosuppressive strategy in patients who may need a surgical management.

Given the retrospective analysis of the first part of this work, our study may be subjected to possible biases and our results should be cautiously generalised. The medical records were evaluated assessing those patients with clear symptoms and underwent the cervical spine diagnostics, including both MRI and CT. Thus, a relatively small number of patients were retrieved with this manifestation which is associated with another weakness of this section. In fact, following generally accepted methodological considerations^14,15^, only univariate explorative analyses were performed in deriving the associated clinical factors with severe atlantoaxial joint involvement in RA patients. In addition, a causal relationship could not be fully established by these explorative analyses since it needs properly conducted prospective studies. Concerning the prospective recruitment that served to build the descriptive cross-sectional evaluation, a limitation of our study is the relatively small number of assessed patients. Furthermore, considering the lack of accurate predictive models about the evolution toward a severe atlantoaxial joint involvement, our descriptive findings could be considered as “hypothesis-generating” to arrange specific powered studies to fully elucidate this unmet need of the management of patients with RA also assessing possible predictive biomarkers^38–40^.

## Conclusions

In conclusion, the occurrence of atlantoaxial joint involvement in RA patients was estimated in a “real-life” setting, possibly causing severe neurologic damage, and requiring a timely management. Male gender, a longer disease duration, presence of ACPA, and extra-articular manifestations resulted to be associated with such manifestation. MRI could provide a useful clinical tool to early evaluate the atlantoaxial joint involvement in RA, also in asymptomatic patients. An early recognition and treatment of such manifestation is of crucial importance to improve the outcome of these patients reducing the consequent associated morbidity.

## Data Availability

All data relevant to the study are included in the article.

## Abbreviations

RA: Rheumatoid arthritis
CT: Computed tomography
MRI: Magnetic resonance imaging
BMI: Body mass index
RF: Rheumatoid factor
ACPA: Anticitrullinated protein antibodies
GCs: Glucocorticoids, -, biological-, and
DMARDs: Disease modifying anti-rheumatic drugs
csDMARDs: Conventional synthetic-disease modifying anti-rheumatic drugs
bDMARDs: Biological-disease modifying anti-rheumatic drugs
tsDMARDs: Target synthetic-disease modifying anti-rheumatic drugs
STIR: Short-tau inversion recovery
SD: Standard deviation
MTX: Methotrexate
ILD: Interstitial lung disease
TNFi: TNF inhibitors
